# Association between the C-reactive protein-triglyceride-glucose index and the presence and prognosis of coronary microvascular dysfunction in patients with chronic coronary syndrome

**DOI:** 10.3389/fcvm.2026.1803811

**Published:** 2026-07-01

**Authors:** Cailin Feng, Jiasuer Alifu, Wen Zhang, Lu Liu, Guoqing Yin, Abdul-Quddus Mohammed, Fuad A. Abdu, Wenliang Che

**Affiliations:** 1 Department of Cardiology, Shanghai Tenth People’s Hospital, School of Medicine, Tongji University, Shanghai, China; 2 Department of Cardiology, Shanghai Tenth People’s Hospital, Chongming Branch, Shanghai, China

**Keywords:** chronic coronary syndromes, clinical outcomes, coronary microvascular dysfunction, C-reactive protein-triglyceride-glucose index, triglyceride-glucose index

## Abstract

**Background:**

Metabolic disturbances in lipid and glucose metabolism underlie coronary microvascular dysfunction (CMD) in chronic coronary syndromes (CCS), yet a practical biomarker integrating this metabolic risk is lacking.

**Methods:**

In this single-center retrospective cohort, 421 CCS patients undergoing coronary angiography were included. CMD was defined by caIMR ≥ 25 U. C-reactive protein-triglyceride-glucose index (CTI), triglyceride-glucose index (TyG), and C-reactive protein (CRP) were measured. Associations with CTI and major adverse cardiovascular events (MACE) in CMD patients were analyzed using Cox regression, mediation, and ROC analyses.

**Results:**

CTI was independently associated with CMD after full adjustment (Q4 vs. Q1: OR: 2.28, 95% CI: 1.16–4.49, *p* = 0.017), outperforming TyG and CRP. Over a median 35-month follow-up, higher CTI predicted increased MACE risk (fully adjusted HR for Q4: 2.62, 95% CI: 1.25–5.51, *p* = 0.011), especially in CMD patients (fully adjusted HR: 2.84, 95% CI: 1.06–7.62, *p* = 0.039). CTI demonstrated a superior predictive accuracy for CMD-related MACE compared with TyG (DeLong test *p* = 0.020).

**Conclusion:**

CTI is a robust and independent predictor of both CMD and MACE in patients with CCS, supporting its use for early risk stratification and personalized management in patients with suspected or confirmed microvascular dysfunction.

## Graphical Abstract

**Figure:**
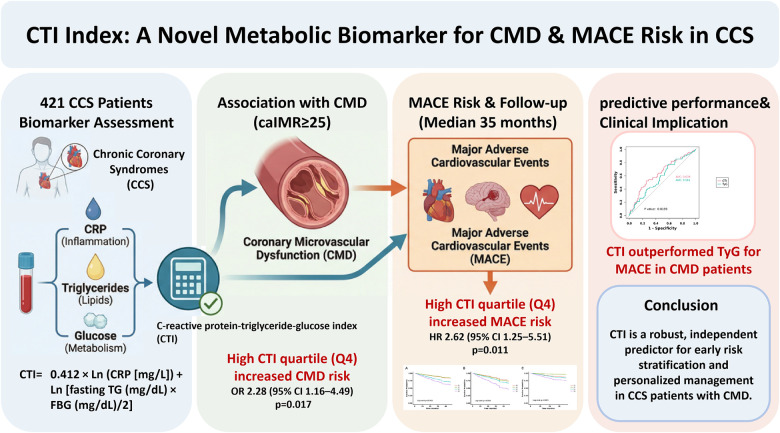

## Introduction

Coronary artery disease (CAD), a leading cause of global morbidity and mortality, is clinically categorized into acute coronary syndromes (ACS) and chronic coronary syndromes (CCS), with CCS constituting the majority of clinical presentations (1). Within CCS, CMD is a critical pathophysiological factor. CMD involves structural and functional abnormalities in the coronary microcirculation, which can significantly contribute to myocardial ischemia (2). Substantial evidence confirms that CMD significantly contributes to myocardial ischemia, angina, and adverse clinical outcomes, even in the absence of significant epicardial coronary stenosis (3). Importantly, CMD is strongly associated with worse prognosis across a range of cardiovascular conditions, including ACS, heart failure with preserved ejection fraction (HFpEF), myocardial infarction with non-obstructive coronary arteries (MINOCA), and CCS itself (4–7). Given its substantial impact on patient health, early prevention and precise risk stratification for CMD are essential to slowing disease progression and improving long-term outcomes.

Insulin resistance (IR), defined as diminished sensitivity and responsiveness to insulin, represents a fundamental metabolic disorder (8). Its significant contribution to CAD pathogenesis involves multiple mechanisms, including disrupted glucose metabolism, enhanced free radical generation, activation of the mitochondrial electron transport chain, and consequent overproduction of reactive oxygen species (ROS) (9). Recognized as a major risk factor, IR is closely implicated in the onset and progression of CMD (10). The TyG index has emerged as a well-validated, sensitive, and specific surrogate biomarker for assessing IR and has demonstrated significant predictive value for CMD (11). However, the TyG index primarily reflects abnormalities in glucose and lipid metabolism and does not comprehensively encompass other key pathological processes closely associated with atherosclerosis and microvascular damage, such as inflammation and oxidative stress.

The CTI is a novel composite biomarker that integrates the TyG index and CRP to provide a multidimensional assessment of cardiovascular risk (12). The prognostic value of CTI has since been established across various clinical contexts, including metabolically heterogeneous populations, patients with cardio-renal-metabolic syndrome, and those with CAD and diabetes (13–15). This emerging parameter precisely encompasses two key factors influencing the development and prognosis of CMD—insulin resistance and inflammation. However, the relationship between CTI and the risk of CMD in patients with CCS remains unexplored.

Therefore, this study was designed to examine the association between the CTI and the risk of CMD in patients with CCS, and to assess the prognostic value of the CTI in CCS patients diagnosed with CMD.

## Methods

### Study design and participants

This single-center, retrospective, observational study consecutively enrolled patients admitted for CCS who underwent coronary angiography (CAG) at Shanghai Tenth People's Hospital between June 2015 and June 2019. Eligible participants were adults (>18 years) with suspected or confirmed CCS, diagnosed in accordance with the 2024 ESC guidelines (1). Key exclusion criteria comprised myocardial infarction within 7 days prior to angiography, history of coronary artery bypass grafting, severe hepatic or renal dysfunction, malignancy, left ventricular ejection fraction <35%, or other severe systemic illnesses. Angiographic images were excluded if they showed poor contrast opacification, significant vascular overlap/distortion of the interrogated artery, or suboptimal quality, as defined in prior methodology (16). Trained physicians, blinded to the study objectives, extracted clinical data from medical records. The study was conducted in compliance with the Declaration of Helsinki and was approved by the Ethics Committee of Shanghai Tenth People's Hospital. Written informed consent was obtained from all enrolled participants.

### Assessment of coronary microvascular dysfunction

All participants underwent assessment of coronary microvascular function via the coronary angiography-derived index of microcirculatory resistance (caIMR). The caIMR analyses were performed offline by trained readers in the catheterization laboratory of Shanghai Tenth People's Hospital who were blinded to clinical outcomes, using the FlashAngio system suite (Rainmed Co., Ltd., Suzhou, China). The theoretical basis and calculation formula for caIMR have been described in detail in prior publications (17). Using the caIMR, we assessed microvascular function in 640 coronary arteries by measuring at all epicardial vessels without significant stenosis and selecting the highest value among all measured arteries.

### Definitions

The CTI index was obtained by using the following formula: CTI = 0.412 × Ln [CRP (mg/L)] + TyG (12). The TyG index was computed as Ln [fasting TG (mg/dL) × FBG (mg/dL)/2] (18). Body mass index (BMI) was calculated as weight in kilograms divided by the square of height in meters (kg/m^2^). CMD was defined as a caIMR value of ≥25 U, based on a previously established cut-off (17).

### Study outcomes and follow-up

The median follow-up duration of our study was 35 months. Follow-up data were collected by trained physicians from Shanghai Tenth People's Hospital via telephone interviews, hospital records, and outpatient visits. The primary endpoint was a composite of MACE, which included cardiovascular death, non-fatal myocardial infarction, heart failure, non-fatal stroke, ischemia-driven revascularization, and angina-related readmission. Cardiovascular death was defined as death attributed to malignant arrhythmia, acute myocardial infarction, heart failure, or other cardiac causes. Non-fatal myocardial infarction was defined based on positive cardiac biomarkers along with typical ischemic symptoms or dynamic electrocardiographic changes. The diagnosis of heart failure was established in accordance with current guidelines (19). Non-fatal stroke was defined as an acute cerebral infarction confirmed by typical clinical symptoms or imaging studies. Ischemia-driven revascularization referred to any revascularization procedure prompted by recurrent angina or objective evidence of cardiac ischemia. Angina-related readmission was defined as any hospital readmission due to angina pectoris requiring medical management, excluding those for revascularization.

### Statistical analysis

Continuous variables are presented as mean ± standard deviation and were compared across groups using Welch's *t*-test or analysis of variance (ANOVA). For variables with a non-normal distribution, data are presented as medians with interquartile ranges. Categorical variables are reported as frequencies and percentages and were compared using the Chi-square test or Fisher's exact test, as appropriate.

Participants were stratified into quartiles based on the CTI value: Q1 (CTI ≤ 8.70), Q2 (8.70 < CTI ≤ 9.10), Q3 (9.10 < CTI ≤ 9.60), and Q4 (CTI > 9.60). Fully adjusted Kaplan–Meier curves with log-rank tests were used to compare MACE-free survival across CTI quartiles (adjusted as model3). Cox proportional-hazards regression models were employed to examine the association between CTI and incident MACE. For comprehensive evaluation, three incremental models were constructed for the overall CCS cohort and separately for patients with or without CMD: Model 1: Unadjusted. Model 2: Adjusted for sex, age, BMI, and Diabetes. Model 3: Additionally adjusted for CMD, low-density lipoprotein cholesterol (LDL-C), hypertension, hyperlipidemia, smoking status, atrial fibrillation, heart failure, and post-PCI. A variance inflation factor (VIF) of less than 5 was considered indicative of no significant multicollinearity in the model (Supplementary Table S1). The proportional hazards assumption was assessed using log-minus-log (LML) plots (Supplementary Figure S1).

Fully adjusted restricted cubic spline (RCS) analysis was performed to explore the dose-response relationship between CTI and MACE risk. The predictive performance of CTI and the TyG index for MACE was assessed using receiver operating characteristic (ROC) curves, and the prognostic value of CTI was evaluated by the area under the curve (AUC) and DeLong test. All analyses were performed using R software (version 4.2.2). A two-sided *p*-value < 0.05 was considered statistically significant.

## Results

### Baseline characteristics

**Figure 1:**
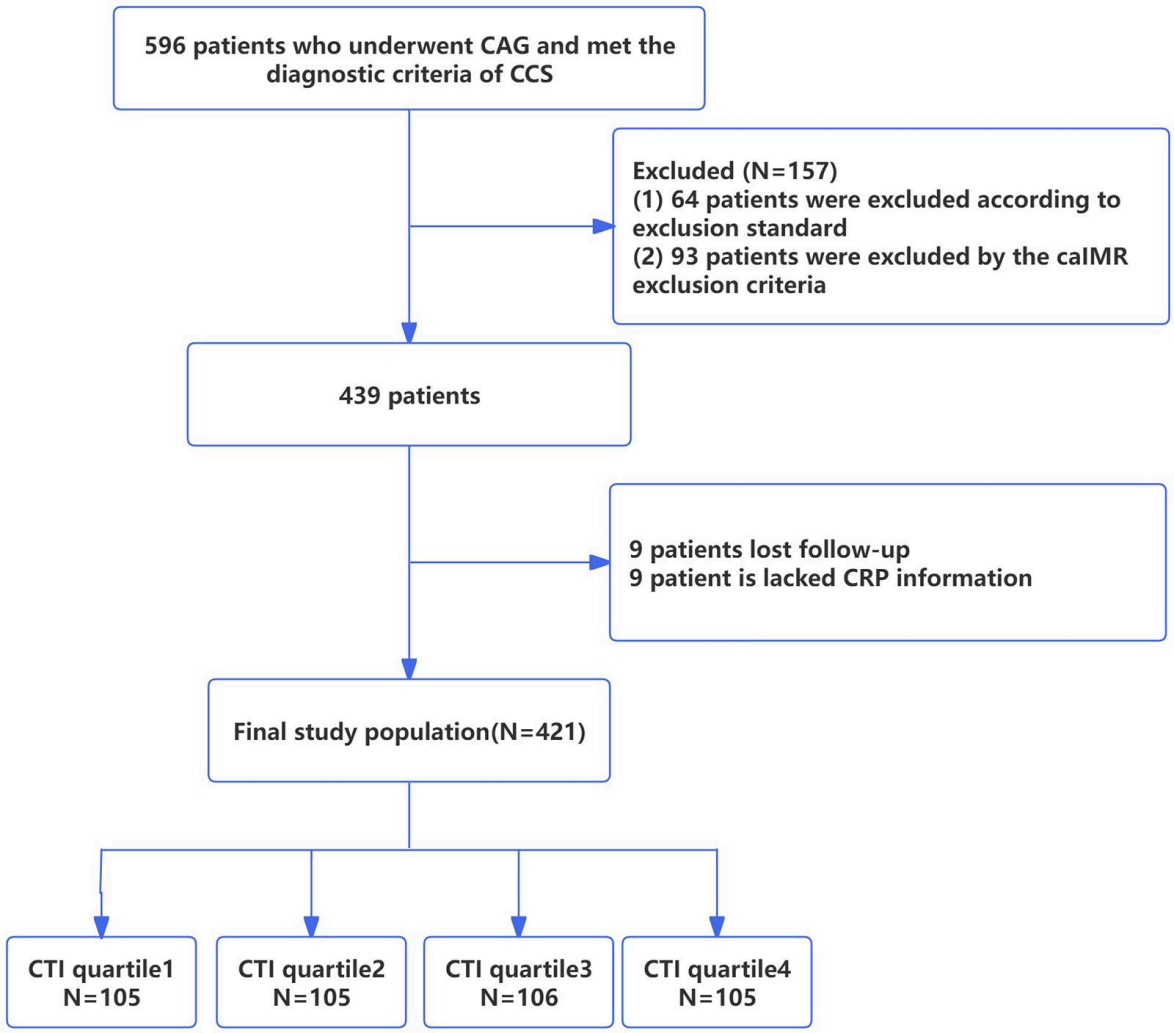
Flow chart of the study population.

A total of 596 patients who underwent coronary angiography and met the diagnostic criteria for CCS were initially enrolled. Of these, 64 patients were excluded based on the predefined exclusion criteria, 93 were excluded due to failure to meet caIMR assessment criteria, 9 were lost to follow-up, and 9 were excluded due to missing key CTI data. Consequently, 421 patients were included in the final analysis (Figure 1).

The baseline characteristics of the 421 participants across the four CTI quartiles are presented in Table 1. The distributions of age, sex, height, and several categorical variables including hypertension, smoking status, history of stroke, atrial fibrillation, heart failure, and whether PCI was performed were comparable across the CTI quartiles (all *p* > 0.05). However, significant differences were observed across groups for multiple cardiometabolic parameters. There was a graded increase across ascending CTI quartiles in body weight, BMI, CaIMR, TyG index, CRP, fasting blood glucose (FBG), triglyceride (TG), total cholesterol (TC), hemoglobin A1c (HbA1c), and the prevalence of CMD, diabetes, and hyperlipemia (all *p* < 0.05). In contrast, high-density lipoprotein-cholesterol (HDL-C) levels were significantly lower in higher CTI quartiles (*p* < 0.001). Diastolic blood pressure (DBP) also showed a significant difference (*p* = 0.029), while systolic blood pressure (SBP) estimated glomerular filtration rate (eGFR) and LDL-C levels did not reach statistical significance (all *p* > 0.05).

**Table 1:** Clinical characteristics of the study population stratified by CTI quartile.

	Overall *N* = 421	Q1 *N* = 105	Q2 *N* = 105	Q3 *N* = 106	Q4 *N* = 105	*P*
Sex						0.806
Male, *n* (%)	248 (58.9%)	66 (62.9%)	58 (55.2%)	62 (58.5%)	62 (59.0%)	
Female, *n* (%)	173 (41.1%)	39 (37.1%)	47 (44.8%)	44 (41.5%)	43 (41.0%)	
Age (years)	64 (57, 70)	63 (58, 70)	66 (59, 70)	63 (56, 70)	64 (57, 68)	0.334
Height (cm)	167 (160, 172)	167 (160, 172)	166 (160, 172)	165 (158, 173)	168 (162, 172)	0.320
Weight (kg)	69.2 ± 11.84	66.5 ± 10.90	67.4 ± 11.98	70.6 ± 12.11	72.4 ± 11.53	<0.001
BMI (kg/m^2^)	24.7 (22.7, 27.1)	23.7 (22.0, 25.7)	24.2 (22.1, 26.7)	25.5 (23.2, 27.7)	25.4 (23.9, 27.3)	<0.001
CaIMR	25.2 (20.9, 34.5)	23.6 (20.7, 30.2)	24.3 (20.3, 33.3)	26.1 (21.2, 35.3)	29.5 (22.4, 38.6)	0.004
CMD, *n* (%)	216 (51.3%)	43 (41.0%)	46 (43.8%)	58 (54.7%)	69 (65.7%)	0.001
CTI	9.1 (8.7, 9.6)	8.3 (8.1, 8.6)	8.9 (8.8, 9.0)	9.3 (9.2, 9.5)	10.0 (9.8, 10.3)	<0.001
TyG	8.7 (8.4, 9.1)	8.2 (8.0, 8.4)	8.5 (8.4, 8.6)	8.9 (8.7, 9.1)	9.4 (9.2, 9.7)	<0.001
CRP (mg/L)	3.0 (2.4, 3.2)	0.9 (0.5, 3.0)	3.0 (2.2, 3.0)	3.0 (3.0, 3.0)	3.2 (3.0, 7.3)	<0.001
FBG (mmol/L)	5.2 (4.8, 6.1)	4.9 (4.5, 5.3)	5.2 (4.7, 5.6)	5.3 (4.9, 6.1)	6.1 (5.2, 8.0)	<0.001
TG (mmol/L)	1.4 (1.1, 2.1)	0.9 (0.8, 1.1)	1.2 (1.1, 1.4)	1.7 (1.4, 2.1)	2.4 (1.9, 3.3)	<0.001
TC (mmol/L)	3.6 (3.0, 4.4)	3.4 (3.0, 4.2)	3.5 (3.0, 4.2)	3.6 (3.1, 4.5)	3.9 (3.3, 4.9)	<0.001
HDL-C (mmol/L)	1.1 (0.9, 1.2)	1.2 (1.0, 1.4)	1.1 (1.0, 1.4)	1.0 (0.9, 1.1)	1.0 (0.8, 1.1)	<0.001
LDL-C (mmol/L)	2.0 (1.5, 2.7)	1.8 (1.5, 2.4)	1.8 (1.4, 2.7)	2.1 (1.5, 2.8)	2.2 (1.6, 2.9)	0.080
HbA1c (%)	6.0 (5.7, 6.5)	5.8 (5.6, 6.1)	5.9 (5.6, 6.3)	6.1 (5.8, 6.7)	6.4 (5.9, 7.8)	<0.001
eGFR	104 ± 27	103 ± 26	104 ± 28	104 ± 28	104 ± 27	0.982
SBP (mmHg)	131 (120, 144)	127 (118, 142)	127 (120, 138)	132 (120, 145)	135 (120, 147)	0.218
DBP (mmHg)	78 (70, 86)	75 (70, 84)	77 (70, 84)	80 (72, 88)	79 (73, 87)	0.029
Hypertension, *n* (%)	262 (62.2%)	60 (57.1%)	64 (61.0%)	65 (61.3%)	73 (69.5%)	0.303
Diabetes, *n* (%)	145 (34.4%)	16 (15.2%)	28 (26.7%)	43 (40.6%)	58 (55.2%)	<0.001
Hyperlipemia, *n* (%)	127 (30.2%)	10 (9.5%)	14 (13.3%)	34 (32.1%)	69 (65.7%)	<0.001
Smoke, *n* (%)	83 (19.7%)	13 (12.4%)	24 (22.9%)	25 (23.6%)	21 (20.0%)	0.156
Stroke, *n* (%)	65 (15.4%)	11 (10.5%)	19 (18.1%)	18 (17.0%)	17 (16.2%)	0.426
Atrial fibrillation, *n* (%)	22 (5.2%)	7 (6.7%)	3 (2.9%)	4 (3.8%)	8 (7.6%)	0.348
Heart failure, *n* (%)	5 (1.2%)	1 (1.0%)	1 (1.0%)	1 (0.9%)	2 (1.9%)	0.940
post-PCI, *n* (%)	144 (34.2%)	38 (36.2%)	28 (26.7%)	43 (40.6%)	35 (33.3%)	0.189

BMI, body mass index; caIMR, coronary angiography-derived index of microcirculatory resistance; CMD, coronary microvascular dysfunction; CTI c-reactive protein-triglyceride-glucose index; TyG, triglyceride-glucose index; CRP, c-reactive protein; FBG, fasting blood glucose; TG, triglyceride; TC, total cholesterol; HDL-C, high-density lipoprotein-cholesterol; LDL-C, low-density lipoprotein-cholesterol; HbA1c, hemoglobin A1c; eGFR, estimated glomerular filtration rate; SBP, systolic blood pressure; DBP, diastolic blood pressure; post-PCI, measurement of caIMR after PCI.

### Association between the CTI index and CMD in CCS patients

Table 2 summarizes the baseline characteristics of the study population by CMD status. The median CTI was significantly higher in the CMD group (9.2, IQR: 8.8–9.7) compared to the non-CMD group (9.0, IQR: 8.6–9.5; *p* < 0.001), with a corresponding significant difference in CTI quartile distribution (*p* = 0.001) where a greater proportion of CMD patients were in the highest quartile (Q4: 31.9% vs. 17.6%). Similarly, the TyG index was higher in the CMD group (8.8, IQR: 8.4–9.2 vs. 8.6, IQR: 8.3–9.0; *p* = 0.001), and its quartile distribution also differed significantly (*p* = 0.009), with more CMD patients in the highest TyG quartile (T4: 31.0% vs. 19.0%). No statistically significant difference was observed in median CRP levels between the groups (*p* = 0.077).

**Table 2:** The distribution of CTI, TyG and CRP index at CMD and non-CMD patients.

	Overall	CMD	Non-CMD	*P*
	*N* = 421	*N* = 216	*N* = 205	
CTI	9.1 (8.7, 9.6)	9.2 (8.8, 9.7)	9.0 (8.6, 9.5)	<0.001
CTI quartile				0.001
Q1	105 (24.9%)	43 (19.9%)	62 (30.2%)	
Q2	105 (24.9%)	46 (21.3%)	59 (28.8%)	
Q3	106 (25.2%)	58 (26.9%)	48 (23.4%)	
Q4	105 (24.9%)	69 (31.9%)	36 (17.6%)	
TyG	8.7 (8.4, 9.1)	8.8 (8.4, 9.2)	8.6 (8.3, 9.0)	0.001
TyG quartile				0.009
T1	106 (25.2%)	46 (21.3%)	60 (29.3%)	
T2	105 (24.9%)	46 (21.3%)	59 (28.8%)	
T3	104 (24.7%)	57 (26.4%)	47 (22.9%)	
T4	106 (25.2%)	67 (31.0%)	39 (19.0%)	
CRP	3.0 (2.4, 3.2)	3.0 (3.0, 3.2)	3.0 (1.2, 3.2)	0.077

CMD, coronary microvascular dysfunction; CTI, c-reactive protein-triglyceride-glucose index; TyG, triglyceride-glucose index; CRP, c-reactive protein.

**Figure 2:**
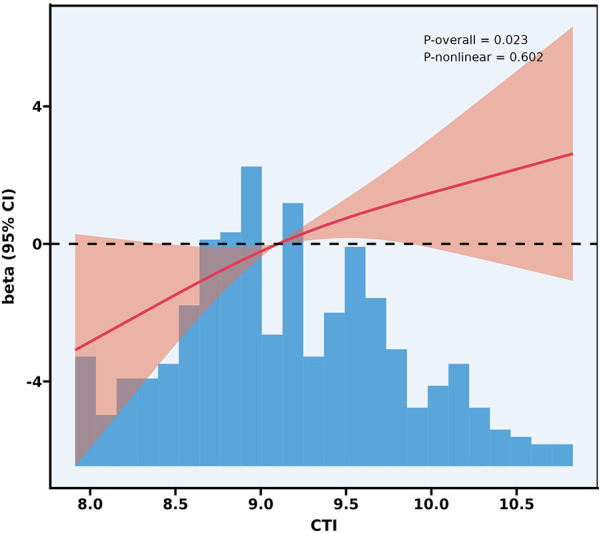
RCS analysis between CTI and caIMR. Adjusted for sex, age, BMI, LDL-C, eGFR, hypertension, diabetes, hyperlipemia, smoke, atrial fibrillation, heart failure, and post-PCI.

The results of the multivariable logistic regression analysis for the association between CTI group and CMD are presented in Table 3. In the unadjusted model (Model 1), a significant positive association was observed. Participants in the highest quartile (Q4) had 2.76 times the odds of CMD compared to those in the lowest quartile (Q1) (OR: 2.76, 95% CI: 1.58 to 4.84, *p* < 0.001). After adjusting for sex, age, BMI, and diabetes (Model 2), the association attenuated but remained significant for Q4 (OR: 2.46, 95% CI: 1.35 to 4.47, *p* = 0.003), while the association for Q3 was no longer statistically significant (OR: 1.52, 95% CI: 0.86 to 2.68, *p* = 0.149). Further adjustment for a comprehensive set of clinical and demographic factors (Model 3) yielded similar results, with a significant association for Q4 (OR: 2.28, 95% CI: 1.16 to 4.49, *p* = 0.017) and a persistent positive trend (*p* = 0.013). In contrast, the association between TyG index quartiles and CMD was not statistically significant in the fully adjusted model (T4, *p* = 0.310; Supplementary Table S2). CRP was not significantly associated with CMD in any model (all *p* > 0.05; Supplementary Table S3). Finally, RCS analysis demonstrated a significant positive linear association between CTI and caIMR (*p* = 0.023, *p*-nonlinear = 0.602; Figure 2).

**Table 3:** Association between CMD and CTI quartile.

	Model 1			Model 2			Model 3		
	OR	95% CI	*p*-value	OR	95% CI	*p*-value	OR	95% CI	*p*-value
CTI quartile									
Q1	Ref			Ref			Ref		
Q2	1.12	0.65, 1.94	0.675	1.07	0.61, 1.87	0.820	1.06	0.60, 1.88	0.843
Q3	1.74	1.01, 3.01	0.046	1.52	0.86, 2.68	0.149	1.46	0.80, 2.65	0.216
Q4	2.76	1.58, 4.84	<0.001	2.46	1.35, 4.47	0.003	2.28	1.16, 4.49	0.017
*P* for trend			<0.001			0.002			0.013

CI, confidence Interval; HR, hazard Ratio.

Model 1: no covariates were adjusted.

Model 2: adjusted for Sex, Age, BMI, and Diabetes.

Model 3: adjusted for Sex, Age, BMI, LDL-C, eGFR, Hypertension, Diabetes, Hyperlipemia, Smoke, Atrial fibrillation, Heart failure, and post-PCI.

### Association between the CTI and clinical outcome

**Figure 3:**
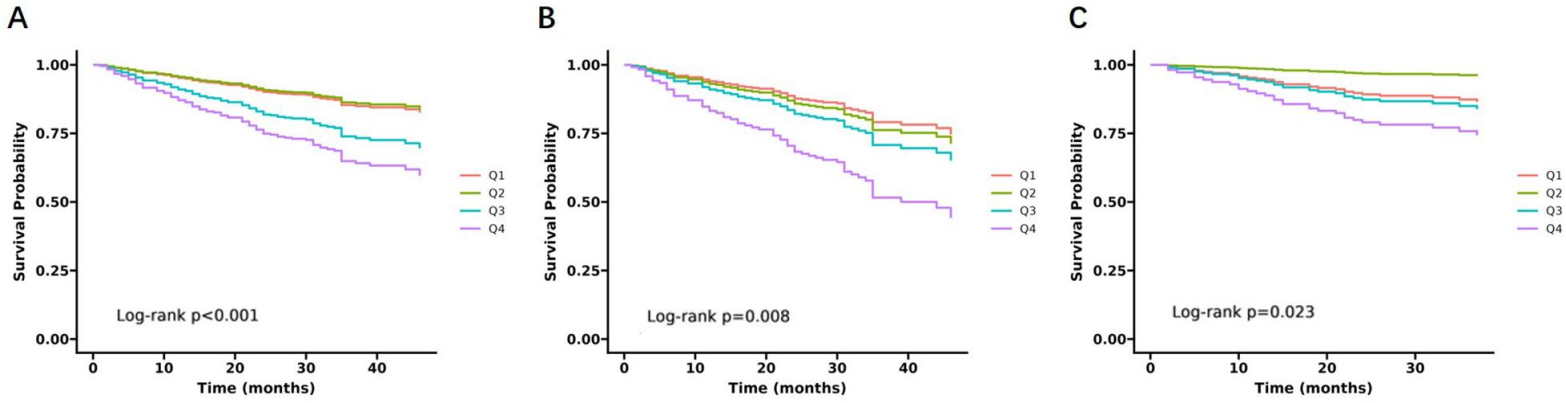
Kaplan–Meier survival curve for MACE in different CTI quartile groups. **(A)** All participants; **(B)** participants with CMD; **(C)** participants with non-CMD. Adjusted for sex, age, BMI, CMD, LDL-C, eGFR, hypertension, diabetes, hyperlipemia, smoke, atrial fibrillation, heart failure, and post PCI.

As shown in Table 4, the baseline characteristics of patients stratified by CTI quartiles revealed a significant association with the primary outcome. The overall incidence of MACE in the cohort of 421 patients was 21.4% (*n* = 90), including 4 (1.0%) cardiovascular death, 4 (1.0%) nonfatal MI, 14 (3.3%) nonfatal stroke, 17 (4.0%) heart failure, 20 (4.8%) ischemia-driven revascularizations, and 31 (7.4%) Angina-related readmission. When analyzed by CTI quartiles, the incidence demonstrated a pronounced and significant ascending trend across quartiles (*p* < 0.001). Specifically, the lowest incidence was observed in Q1 (13.3%, *n* = 14) and Q2 (13.3%, *n* = 14) quartiles. The incidence increased markedly in Q3 (24.5%, *n* = 26) and was highest in Q4 (34.3%, *n* = 36). Kaplan–Meier curves demonstrated that the risk of MACE was consistently highest in the Q4 group across the overall population (log-rank *p* < 0.001; Figure 3A), as well as in both the CMD (log-rank *p* = 0.008; Figure 3B) and non-CMD subgroups (log-rank *p* = 0.023; Figure 3C).

**Table 4:** Clinical outcomes of CCS patients according to CTI level.

	Overall *N* = 421	Q1 *N* = 105	Q2 *N* = 105	Q3 *N* = 106	Q4 *N* = 105	*P*
MACE	90 (21.4%)	14 (13.3%)	14 (13.3%)	26 (24.5%)	36 (34.3%)	<0.001
Cardiovascular death	4 (1.0%)	0	1 (1.0%)	1 (0.9%)	2 (1.9%)	
Nonfatal MI	4 (1.0%)	0	0	2 (1.9%)	2 (1.9%)	
Nonfatal stroke	14 (3.3%)	2 (1.9%)	2 (1.9%)	4 (3.8%)	6 (5.7%)	
Heart failure	17 (4.0%)	3 (2.9%)	2 (1.9%)	5 (4.7%)	7 (6.7%)	
Ischemia-driven revascularization	20 (4.8%)	3 (2.9%)	4 (3.8%)	5 (4.7%)	8 (7.6%)	
Angina-related readmission	31 (7.4%)	6 (5.7%)	5 (4.8%)	9(8.5%)	11(10.5%)	

MACE, major adverse cardiovascular event; MI, myocardial infarction.

To evaluate the association between CTI and MACE risk, three Cox proportional hazards models were constructed. Table 5 presents the Cox regression analyses of CTI quartiles (Q1 as reference) and MACE risk in CCS patients, overall and stratified by CMD status. In the overall cohort, a significant dose-response trend was observed across all models (*p*-trend ≤ 0.002). While the association for Q3 attenuated after full adjustment (*p* = 0.095), the highest quartile (Q4) remained independently associated with increased MACE risk (HR: 2.62, 95% CI: 1.25–5.51, *p* = 0.011). This association was more pronounced in patients with CMD, where Q4 exhibited significantly elevated hazard ratios across all models (fully adjusted HR: 2.84, 95% CI: 1.06–7.62, *p* = 0.039), with consistent positive trends (*p*-trend = 0.016). In contrast, among non-CMD patients, no statistically significant associations were observed between higher CTI quartiles and MACE in any models. For the TyG index, although T4 showed strong unadjusted associations with increased MACE risk in CMD patients (Model 1, HR: 2.31, 95% CI: 1.04–5.15; *p* = 0.040; Supplementary Table S4), these associations were no longer significant in the fully adjusted model (Model 3, HR: 2.38, 95% CI: 0.82–6.91, *p* = 0.111; Supplementary Table S4), and the dose-response trend was abolished (*p*-trend = 0.118). CRP was not significantly associated with MACE in any model (all *p* > 0.05; Supplementary Table S5).

**Table 5:** Cox regression analysis for MACE according to CTI quartile.

	Model 1			Model 2			Model 3		
	HR	95% CI	*p*-value	HR	95% CI	*p*-value	HR	95% CI	*p*-value
All CCS patients									
Q1	Ref			Ref			Ref		
Q2	0.97	0.46, 2.04	0.936	0.97	0.46, 2.04	0.931	0.88	0.41, 1.88	0.741
Q3	1.95	1.02, 3.74	0.043	2.05	1.05, 4.03	0.036	1.82	0.90, 3.66	0.095
Q4	2.83	1.53, 5.26	<0.001	3.09	1.60, 5.95	<0.001	2.62	1.25, 5.51	0.011
*P* for trend			<0.001			<0.001			0.002
CMD patients									
Q1	Ref			Ref			Ref		
Q2	1.21	0.52, 2.80	0.656	1.05	0.44, 2.53	0.907	0.84	0.31, 2.30	0.735
Q3	1.46	0.64, 3.33	0.370	1.51	0.64, 3.59	0.349	1.48	0.58, 3.76	0.412
Q4	2.89	1.39, 6.00	0.004	2.75	1.26, 6.00	0.011	2.84	1.06, 7.62	0.039
*P* for trend			0.002			0.002			0.016
Non-CMD patients									
Q1	Ref			Ref			Ref		
Q2	0.33	0.07, 1.63	0.174	0.34	0.07, 1.68	0.184	0.88	0.26, 2.98	0.842
Q3	1.34	0.46, 3.85	0.591	1.59	0.53, 4.77	0.409	2.57	0.83, 7.98	0.102
Q4	2.29	0.87, 6.02	0.094	2.71	0.99, 7.41	0.053	2.67	0.75, 9.53	0.131
*P* for trend			0.021			0.011			0.047

CI, confidence Interval; HR, hazard Ratio.

Model 1: no covariates were adjusted.

Model 2: adjusted for sex, age, BMI, and diabetes.

Model 3: adjusted for sex, age, BMI, CMD, LDL-C, eGFR, hypertension, diabetes, hyperlipemia, smoke, atrial fibrillation, heart failure, and post-PCI.

**Figure 4:**
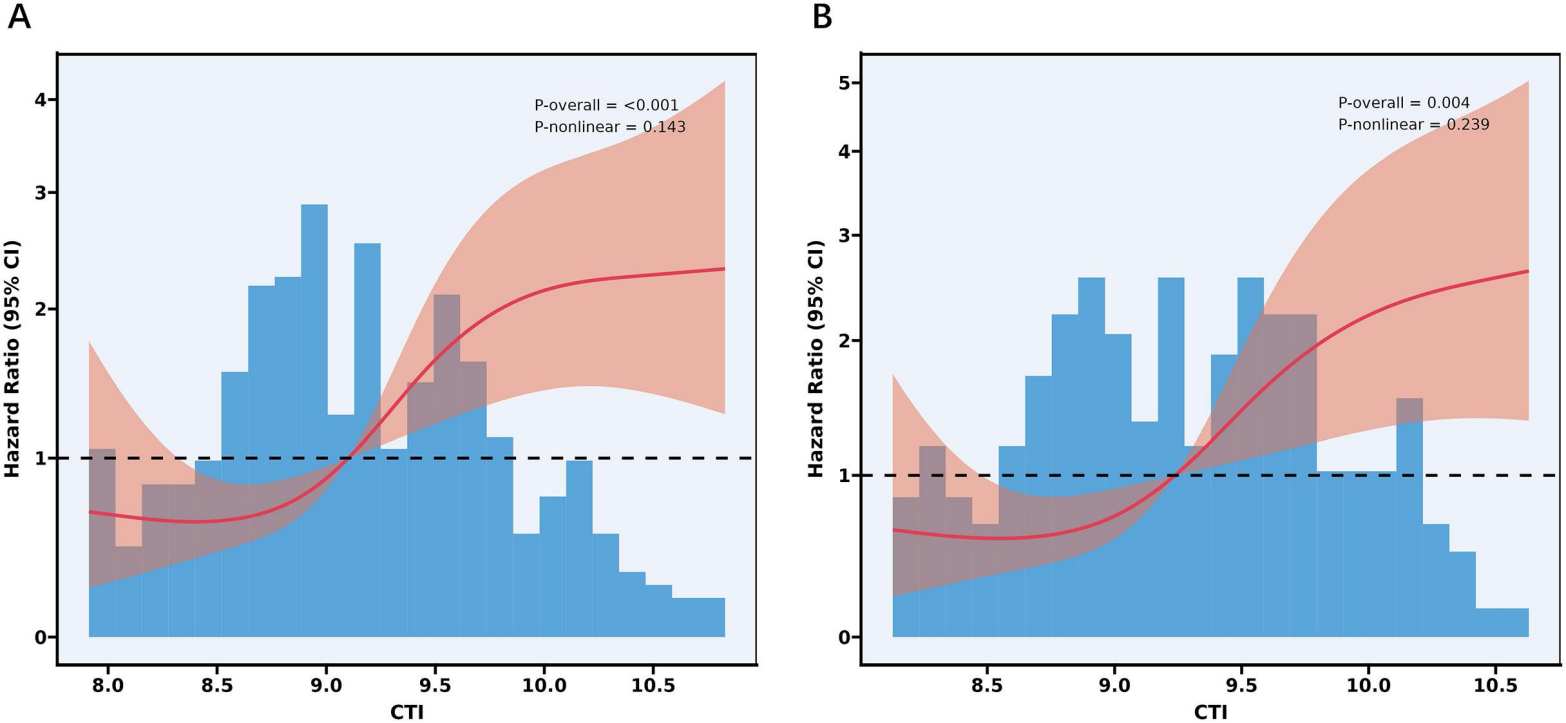
RCS analysis between CTI and MACE. **(A)** All participants; **(B)** participants with CMD. Adjusted for sex, age, BMI, CMD, LDL-C, eGFR, hypertension, diabetes, hyperlipemia, smoke, atrial fibrillation, heart failure, and post PCI.

**Figure 5:**
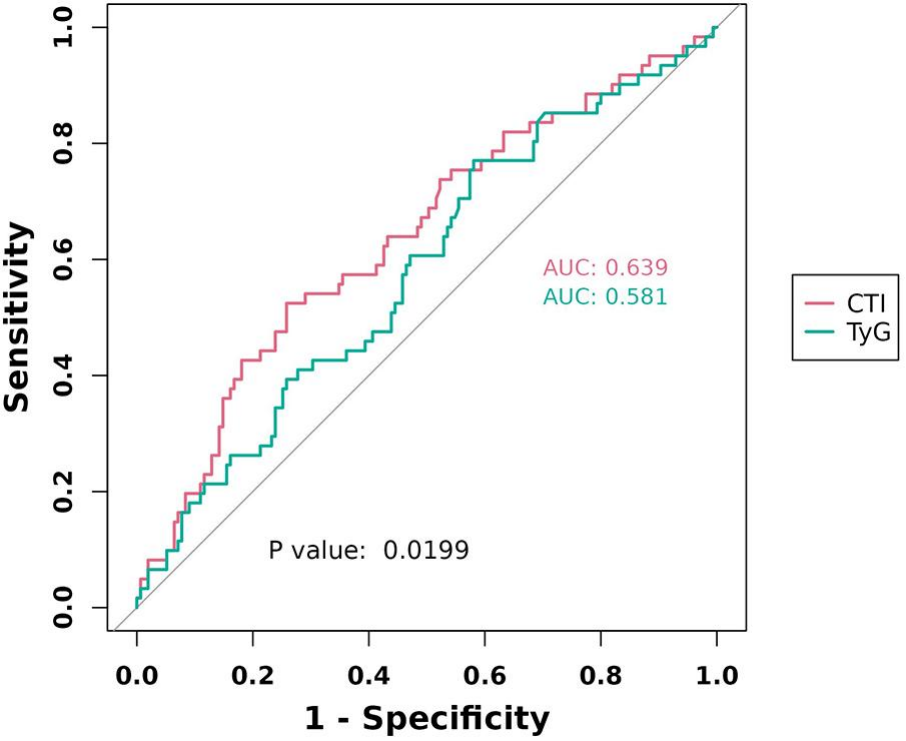
Predictive performance of CTI, TyG index for MACE in patients with CMD.

RCS curves revealed a significant linear relationship between CTI and prognosis in the overall population (*p* < 0.001, *p*-nonlinear = 0.143; Figure 4A) and patients with CMD (*p* = 0.004, *p*-nonlinear = 0.239; Figure 4B). ROC curve analysis indicated that CTI exhibited the higher predictive accuracy for incident MACE in CMD patients than TyG (AUC: 0.64 vs. 0.58, Delong test *p* = 0.020, Figure 5).

## Discussion

To our knowledge, the present study is the first to systematically evaluate the association between the CTI index and both the presence of CMD and long-term prognosis in patients with CCS. In this retrospective cohort study, we enrolled 421 CCS participants, among whom 216 were diagnosed with CMD. Our analyses revealed that the CTI—a composite biomarker integrating inflammatory status and metabolic dysfunction—was significantly elevated in patients with CMD compared to those without CMD. After comprehensive adjustment for demographic characteristics, lifestyle factors, and clinical risk factors (Model 3), CTI was associated with a markedly higher risk of CMD than either the TyG index or CRP alone. Furthermore, the predictive performance of CTI for MACE in CCS patients with CMD outperformed TyG index. Notably, RCS analyses consistently demonstrated a linear dose-response relationship between CTI levels and MACE hazard ratios in CMD patients.

The pathophysiology of CCS is multifaceted and not fully explained by epicardial coronary artery disease alone, which frequently fails to account for the clinical presentation and adverse prognosis observed in affected individuals (1). A significant proportion of patients with CCS continue to suffer from typical angina and experience poor outcomes, even in the absence of obstructive stenosis or after complete revascularization (20). Consequently, increasing focus has been placed on the adverse contribution of CMD, which may provide pivotal insights for modifying risk in CCS. Defined by structural and functional abnormalities in the coronary microcirculation, CMD leads to impaired myocardial perfusion. A central mechanism in its pathogenesis is oxidative stress, which compromises nitric oxide-mediated vasodilation and promotes the progression of CMD (21). These microcirculatory impairments can induce myocardial ischemia, thereby producing clinical symptoms such as angina. Robust evidence confirms that CMD is prevalent in CCS populations, predicts a worse prognosis, and is commonly associated with diminished quality of life, impaired mental health, and reduced exercise capacity (22, 23). Recognition of its clinical significance has grown substantially.

This heightened awareness has considerably elevated the importance of CMD research. The evaluation of CMD has been advanced by the development of caIMR, a reliable, non-invasive metric with high diagnostic accuracy that eliminates the requirement for specialized wires or hyperemic agents (5, 17). Elevated caIMR has been linked to adverse cardiac events in STEMI (24), in-hospital complications in Takotsubo syndrome (25), and increased risks of cardiac death and heart failure-related readmission in INOCA patients (5). Furthermore, caIMR carries prognostic value for predicting MACE in patients across the CCS spectrum, including those with MINOCA, HFpEF, diabetes, or obesity (6, 26, 27). In our study, CMD was present in 51.3% of CCS patients, among whom 62 experienced MACE (Supplementary Table S6), confirming its high prevalence and the associated morbidity and mortality in this cohort. These findings highlight the imperative for early detection and diagnosis of CMD, as well as the urgent need to develop effective risk-stratification strategies to improve the generally unfavorable prognosis linked to CCS.

Both inflammation and IR can reduce the production of vasoprotective Nitric oxide (NO) by diminishing endothelial NO synthase activity, thereby contributing to CMD (28). Additionally, hyperinsulinemia may promote CMD by enhancing oxidative stress and the release of pro-inflammatory cytokines (29). The TyG index has emerged as a practical surrogate marker for IR, an elevated TyG index is linked to various cardiovascular disease (CVD) manifestations, including symptomatic CAD, arterial stiffness, coronary artery calcification, in-stent restenosis, and multi-vessel CAD (30–34). While CRP is a well-established biomarker of chronic low-grade inflammation, a recent meta-analysis confirms that elevated levels of inflammatory markers such as CRP, fibrinogen, Galectin-3 and IL-6, are strongly associated with CVD incidence in general populations (35). The CTI, which combines the TyG index and CRP, was developed by Ruan et al. (12) and serves as a significant tool for prognostic evaluation in CVD. Earlier research using NHANES data demonstrated that elevated CTI levels are associated with both increased CVD incidence and mortality (36).

We therefore hypothesize that CTI, by integrating both metabolic and inflammatory components, may provide stronger or more consistent predictive value for CMD risk in CCS patients than either component alone. In our study, CTI was significantly higher in CMD patients than in non-CMD patients. Furthermore, CTI demonstrated better association for CMD risk than TyG or CRP alone and exhibited a linear association with CMD incidence. These findings align with previous reports of an indirect relationship between IR, inflammation, and CMD, highlighting the potential need for interventions targeting IR and systemic inflammation to prevent CMD in CCS patients. The association between CTI and CMD may stem from its ability to comprehensively capture the intertwined metabolic-inflammatory state central to cardiovascular pathogenesis. While the TyG index and CRP individually reflect insulin resistance and systemic inflammation, respectively, their integration in the CTI accounts for potential interactions between these pathways and reduces misclassification due to individual biomarker variability. This composite approach provides a more robust assessment of the underlying pathophysiological milieu, thereby improving the identification of high-risk individuals.

Numerous studies have demonstrated that the CTI index can predict adverse outcomes in patients with CVD (13, 36). Ding, Wenlong et al. reported a nonlinear relationship between CTI and all-cause mortality risk in CVD patients, with elevated CTI levels being significantly associated with increased mortality (37). Furthermore, elevated CTI has been identified as a robust and independent predictor of all-cause and cardiovascular mortality in elderly populations in both the USA and China (38). Another study by Gao, Ang et al. indicated that CTI is a practical indicator for evaluating cardiometabolic diseases and represents a promising index for predicting recurrent cardiovascular risk in patients undergoing PCI (39). Extending this evidence, our study specifically establishes the robust prognostic value of CTI in patients with CMD.

Our findings indicate that the CTI index is not only associated with the presence of CMD but also remains significantly correlated with worse clinical outcomes after adjustment for confounding risk factors. This underscores the importance of IR and inflammatory metabolism, as captured by the CTI index, in the pathogenesis of CMD. Moreover, CTI consistently demonstrated better predictive performance for MACE than TyG, exhibiting a higher AUC and revealing a significant linear association with MACE in CCS patients with CMD. Our study provides significant clinical evidence for the predictive value of the CTI index regarding MACE occurrence in CMD patients. As a simple, readily obtainable measure, the CTI index exhibits favorable clinical applicability for risk stratification in CMD, which may facilitate early identification of high-risk patients and guide more personalized treatment decisions in clinical practice.

### Study limitations

Our study has several potential limitations. First, the study is limited by its single-center, retrospective, observational design and a relatively modest sample size. Validation of these findings will require larger-scale, prospective, multi-center studies. Second, although a wide range of confounders was adjusted for in the analysis, residual confounding may persist due to unmeasured variables—such as physical activity, dietary habits, subclinical conditions, or genetic predisposition—that could influence both CTI and the risk of MACE. Third, caIMR, as a relatively novel surrogate technique, remains operator-dependent to some degree. Moreover, participants in this study did not undergo invasive IMR assessment for CMD. Fourth, TG, FBG, and CRP levels were measured only at admission; their fluctuations during follow-up were not captured, leaving any potential changes in the CTI index over time unknown. Fifth, the generalizability of our findings may be limited, as the study population consisted exclusively of middle-aged and older Chinese adults. Consequently, these results may not be fully applicable to younger individuals or populations with differing ethnic or socioeconomic backgrounds. Finally, although CTI demonstrated superior predictive performance compared to the TyG index, its discriminative ability—as reflected by the AUC—remained modest. Therefore, CTI alone is insufficient to guide clinical decision-making and should be integrated with established risk scores and biomarkers to improve predictive accuracy.

## Conclusion

This study identifies the CTI index as a robust and independent predictor of both CMD and MACE in patients with chronic coronary syndrome. We demonstrate a significant dose-response relationship, where higher CTI levels are strongly associated with increased prevalence of CMD and a 2.6-fold elevated MACE risk, particularly among patients with established CMD. Notably, CTI's predictive value persists after comprehensive adjustment for metabolic and clinical confounders, outperforming TyG index. Consequently, CTI may serve as a practical clinical tool for early identification and risk stratification of CMD, thereby enabling more targeted monitoring and personalized management in patients with chronic coronary syndrome.

## Data Availability

The original contributions presented in the study are included in the article/Supplementary Material, further inquiries can be directed to the corresponding author/s.
