# Supplementary material for: Association between the C-reactive protein-triglyceride-glucose index and the presence and prognosis of coronary microvascular dysfunction in patients with chronic coronary syndrome
Source: Front Cardiovasc Med. 2026 Jul 1;13:1803811. doi: 10.3389/fcvm.2026.1803811 (PMC13371164; doi:10.3389/fcvm.2026.1803811)
Supplement: Supplementary file 1 [file Datasheet1.docx]

Supplementary Table

**Table S1. Variance Inflation Factor and Tolerance**

| **Variables** | **Tolerance** | **VIF** |
| --- | --- | --- |
| Sex | 0.815 | 1.227 |
| Age | 0.883 | 1.132 |
| BMI | 0.873 | 1.146 |
| LDL-C | 0.834 | 1.200 |
| eGFR | 0.928 | 1.078 |
| Hypertension | 0.962 | 1.039 |
| Diabetes | 0.777 | 1.287 |
| Hyperlipemia | 0.637 | 1.569 |
| Smoke | 0.836 | 1.196 |
| Heart failure | 0.972 | 1.029 |
| Atrial fibrillation | 0.962 | 1.040 |
| post PCI | 0.939 | 1.064 |
| TyG | 0.569 | 1.757 |
| CRP | 0.961 | 1.041 |

**Table S2. Association between CMD and TyG quartile**

|  | **Model 1** | | | **Model 2** | | | **Model 3** | | |
| --- | --- | --- | --- | --- | --- | --- | --- | --- | --- |
|  | **OR** | **95% CI** | **p-value** | **OR** | **95% CI** | **p-value** | **OR** | **95% CI** | **p-value** |
| **TyG quartile** |  |  |  |  |  |  |  |  |  |
| T1 | Ref |  |  | Ref |  |  | Ref |  |  |
| T2 | 1.02 | 0.59, 1.75 | 0.952 | 0.94 | 0.54, 1.64 | 0.834 | 0.91 | 0.51, 1.61 | 0.745 |
| T3 | 1.58 | 0.92, 2.73 | 0.099 | 1.35 | 0.77, 2.37 | 0.301 | 1.28 | 0.71, 2.31 | 0.407 |
| T4 | 2.24 | 1.29, 3.89 | 0.004 | 1.86 | 1.02, 3.38 | 0.041 | 1.46 | 0.71, 3.00 | 0.310 |
| P for trend |  |  | 0.001 |  |  | 0.021 |  |  | 0.764 |
| Abbreviations: CI = Confidence Interval, OR = Odds Ratio | | | | | | | | | |
| Model 1: no covariates were adjusted  Model 2: adjusted for Sex, Age, BMI, and Diabetes  Model 3: adjusted for Sex, Age, BMI, CRP, LDL-C, eGFR, Hypertension, Diabetes, Hyperlipemia, Smoke, Atrial fibrillation, Heart failure, and post-PCI | | | | | | | | | |

**Table S3. Association between CMD and CRP**

|  | **Model 1** | | | **Model 2** | | | **Model 3** | | |
| --- | --- | --- | --- | --- | --- | --- | --- | --- | --- |
|  | **OR** | **95% CI** | **p-value** | **OR** | **95% CI** | **p-value** | **OR** | **95% CI** | **p-value** |
| **CRP** | 1.02 | 0.99, 1.05 | 0.191 | 1.02 | 0.99, 1.06 | 0.184 | 1.02 | 0.99, 1.06 | 0.167 |
| Abbreviations: CI = Confidence Interval, OR = Odds Ratio | | | | | | | | | |
| Model 1: no covariates were adjusted  Model 2: adjusted for Sex, Age, BMI, and Diabetes  Model 3: adjusted for Sex, Age, BMI, TyG, LDL-C, eGFR, Hypertension, Diabetes, Hyperlipemia, Smoke, Heart failure, Atrial fibrillation, and post-PCI | | | | | | | | | |

**Table S4. Cox regression analysis for MACE of CMD patients according to TyG quartile**

|  | **Model 1** | | | **Model 2** | | | **Model 3** | | |
| --- | --- | --- | --- | --- | --- | --- | --- | --- | --- |
|  | **HR** | **95% CI** | **p-value** | **HR** | **95% CI** | **p-value** | **HR** | **95% CI** | **p-value** |
| **TyG** | 1.46 | 1.03, 2.06 | 0.032 | 1.41 | 0.98, 2.03 | 0.063 | 1.45 | 0.89, 2.36 | 0.135 |
| **TyG quartile** |  |  |  |  |  |  |  |  |  |
| T1 | Ref |  |  | Ref |  |  | Ref |  |  |
| T2 | 1.92 | 0.85, 4.34 | 0.118 | 1.84 | 0.79, 4.26 | 0.155 | 1.41 | 0.54, 3.65 | 0.483 |
| T3 | 2.17 | 0.97, 4.83 | 0.058 | 2.48 | 1.07, 5.76 | 0.035 | 1.61 | 0.64, 4.02 | 0.309 |
| T4 | 2.31 | 1.04, 5.15 | 0.040 | 2.40 | 1.01, 5.71 | 0.047 | 2.38 | 0.82, 6.91 | 0.111 |
| P for trend |  |  | 0.043 |  |  | 0.041 |  |  | 0.118 |
| Abbreviations: CI = Confidence Interval, HR = Hazard Ratio | | | | | | | | | |
| Model 1: no covariates were adjusted  Model 2: adjusted for Sex, Age, BMI, and Diabetes  Model 3: adjusted for Sex, Age, BMI, CRP, LDL-C, eGFR, Hypertension, Diabetes, Hyperlipemia, Smoke, Atrial fibrillation, Heart failure, and post-PCI | | | | | | | | | |

**Table S5. Cox regression analysis for MACE and CRP of CMD patients**

|  | **Model 1** | | | **Model 2** | | | **Model 3** | | |
| --- | --- | --- | --- | --- | --- | --- | --- | --- | --- |
|  | **HR** | **95% CI** | **p-value** | **HR** | **95% CI** | **p-value** | **HR** | **95% CI** | **p-value** |
| **CRP** | 1.01 | 0.99, 1.03 | 0.458 | 1.01 | 0.99, 1.03 | 0.390 | 1.01 | 0.99, 1.04 | 0.426 |
| Abbreviations: CI = Confidence Interval, HR = Hazard Ratio | | | | | | | | | |
| Model 1: no covariates were adjusted  Model 2: adjusted for Sex, Age, BMI, and Diabetes  Model 3: adjusted for Sex, Age, BMI, TyG, LDL-C, eGFR, Hypertension, Diabetes, Hyperlipemia, Smoke, Atrial fibrillation, Heart failure, and post-PCI | | | | | | | | | |

**TableS6. Clinical outcomes of CCS patients according to CMD groups**

|  | **Overall** | **CMD** | **Non-CMD** | ***P*** |
| --- | --- | --- | --- | --- |
|  | **N = 421** | **N=216** | **N=205** |  |
| **MACE** | 90 (21.4%) | 62 (28.7%) | 28 (13.7%) | <0.001 |
| Cardiovascular death | 4 (1.0%) | 3 (1.4%) | 1 (0.5%) |  |
| Nonfatal MI | 4 (1.0%) | 4 (1.9%) | 0 |  |
| Nonfatal stroke | 14 (3.3%) | 9 (4.2%) | 5 (2.4%) |  |
| Heart failure | 17 (4.0%) | 11 (5.1%) | 6 (2.9%) |  |
| Ischemia-driven revascularization | 20 (4.8%) | 12 (5.6%) | 8 (3.9%) |  |
| Angina-related readmission | 31 (7.4%) | 22 (10.2%) | 9 (4.4%) |  |

**Abbreviations:** MACE major adverse cardiovascular event, MI myocardial infarction, CMD coronary microvascular dysfunction

Supplementary Figure

**
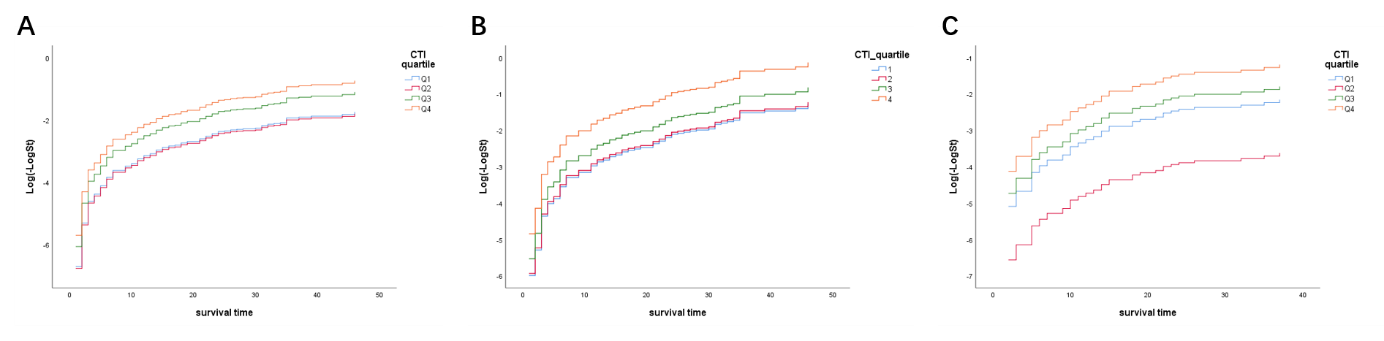
**

**Figure S1.** Log minus log graph for the Cox model3. A: All CCS population; B: CMD population; C: non-CMD population
